# Follicular lymphoma patients with KIR2DL2 and KIR3DL1 and their ligands (HLA-C1 and HLA-Bw4) show improved outcome when receiving rituximab

**DOI:** 10.1186/s40425-019-0538-8

**Published:** 2019-03-12

**Authors:** Amy K. Erbe, Wei Wang, Lakeesha Carmichael, Anna Hoefges, Bartosz Grzywacz, Patrick K. Reville, Erik A. Ranheim, Jacquelyn A. Hank, KyungMann Kim, Songwon Seo, Eneida A. Mendonca, Yiqiang Song, Vaishalee P. Kenkre, Fangxin Hong, Randy D. Gascoyne, Elisabeth Paietta, Sandra J. Horning, Jeffrey S. Miller, Brad Kahl, Paul M. Sondel

**Affiliations:** 1 Department of Human Oncology, University of Wisconsin, Madison, WI USA; 2 Department of Biostatistics and Medical Informatics, University of Wisconsin, Madison, WI USA; 3 Department of Laboratory Medicine and Pathology, University of Minnesota, Minneapolis, MN USA; 4 Department of Medicine, University of Wisconsin, Madison, WI USA; 5 Department of Biostatistics, Harvard University, Dana Farber Cancer Institute, Boston, MA USA; 6 Department of Pathology and Laboratory Medicine, Centre for Lymphoid Cancer, British Columbia Cancer Agency, Vancouver, British Columbia Canada; 7 Montefiore Medical Center-North Division, Albert Einstein College of Medicine, Bronx, NY USA; 8 Department of Medicine, Stanford University, Stanford, CA USA; 9 Department of Medicine, University of Minnesota, Minneapolis, MN USA; 10 Department of Medicine, Washington University School of Medicine, St. Louis, MO USA; 11 Department of Pediatrics, University of Wisconsin, Madison, WI USA; 12 University of Wisconsin-Madison, 1111 Highland Avenue, 4159 WIMR Bldg, Madison, WI 53705 USA

**Keywords:** KIR, HLA, Follicular lymphoma, NK cells, Rituximab, Immunotherapy, Monoclonal antibody, ADCC, CD20, MHC class I

## Abstract

**Background:**

The ECOG-ACRIN Cancer Research Group evaluated rituximab treatment schedules for patients with newly-diagnosed low-tumor-burden follicular-lymphoma (FL). All patients received 4-weekly rituximab treatments as induction therapy. Clinically-responding patients were randomized to receive rituximab every 13 weeks (“maintenance”) vs. no additional rituximab until progression (“non-maintenance”). Based on “time-to-rituximab-failure (TTRF)”, the study-committee reported there was no overall-benefit for maintenance rituximab in this setting. Tumor-reactive mAbs, like rituximab, trigger natural killer (NK) cells. NK-cell responses are regulated, in part, by interactions between killer immunoglobulin-like receptors (KIRs) on NK cells and their interactions with KIR-ligands. In a separate study of children with neuroblastoma treated with a different mAb, we found certain KIR/KIR-ligand genotypes associated with improved outcome. Here, we assessed whether a subset of FL patients show improved outcome from the maintenance rituximab based on these same KIR/KIR-ligand genotypes.

**Methods:**

Genotypes for KIR/KIR-ligand were determined and assessed for associations with outcome [duration of response, TTRF and % tumor shrinkage] as a post-hoc analysis of this phase III trial. Our primary objective was to assess specific KIR/KIR-ligand genotype associations, followed by separate prespecified KIR/KIR-ligand genotype associations in follow-up analyses. Statistical analyses for association of genotype with clinical outcome included: Log-rank tests and Cox proportional hazards regression models to assess duration of response and TTRF; analysis of variance (ANOVA) was used for assessment of % tumor shrinkage.

**Results:**

We found that patients inheriting KIR2DL2 and its ligand (HLA-C1) along with KIR3DL1 and its ligand (HLA-Bw4) had improved outcome over patients without this genotype. In addition, patients with KIR2DL2 and HLA-C1 along with KIR3DL1 and HLA-Bw4 also showed improved duration of response and tumor shrinkage if they received maintenance, while patients without this genotype showed no such improvement when receiving maintenance.

**Conclusions:**

The data presented here indicate that a subset of FL patients, identified by certain KIRs/KIR-ligands, have improved outcome and may benefit from additional rituximab treatment. Taken together, this suggests that the efficacy of tumor-reactive mAb treatment for some patients is influenced by KIRs on NK cells. However, prior to considering these genotypes in a clinically-actionable manner, these findings need independent validation in other studies.

**Electronic supplementary material:**

The online version of this article (10.1186/s40425-019-0538-8) contains supplementary material, which is available to authorized users.

## Background

The most common form of indolent lymphoma is follicular lymphomas (FL). The use of rituximab to treat FL has markedly transformed the care of these patients [1, 2]. For patients with low-tumor burden FL, a maintenance rituximab strategy has been shown to improve the progression-free survival following induction with either chemotherapy or rituximab [3–5]. Yet, whether other clinical outcome parameters could benefit from the continual rituximab treatment schedule (maintenance rituximab vs. a close “watch and wait” approach) was unclear [6]. Additionally, concerns related to the added healthcare expenditures required for a maintenance treatment schedule suggested that it may be more cost-efficient to treat with rituximab on a less frequent basis [6–8]. As such, the ECOG-ACRIN Cancer Research Group (ECOG-ACRIN) conducted and reported results from a phase III clinical trial (E4402) to determine the optimal rituximab dosing strategy for patients with low-tumor burden FL [6]. In this report of the E4402 trial, Kahl and colleagues concluded that maintenance rituximab treatment (continual doses of rituximab every 13 weeks) provided no benefit in the time to rituximab failure for this population of low-tumor burden FL patients, compared to a non-maintenance rituximab treatment schedule (an additional course of 4 weekly doses of rituximab only upon disease progression) [6]. The purpose of this present study was to determine if inherited genotypic variances in genes that influence immune function, and potentially rituximab’s antitumor effects, may identify subpopulations of patients that differ in their outcome following maintenance vs. non-maintenance rituximab schedules [8, 9].

In a separate study of FL patients, patients with lower NK cell counts had inferior clinical prognosis [10]. This result suggests that NK cell count may be used as a prognostic biomarker for FL patients, and that treatments designed to activate NK cells might potentially be beneficial [10]. NK cells contribute to the anti-tumor effects of rituximab via antibody-dependent cell-mediated cytotoxicity (ADCC), and several studies have assessed NK cell specific immunogenetic factors that may be predictive of response to rituximab treatment in FL patients [11–15]. We hypothesize that NK cell specific immunogenetic factors influence the clinical outcome following rituximab treatment for some FL patients, and that the maintenance rituximab treatment schedule differentially impacts clinical outcome dependent upon individual genotypic differences.

NK cell activation is based on the balance of inhibitory and activating signals transmitted by receptors on NK cells. One class of these receptors is killer-cell immunoglobulin-like receptors (KIRs); some of these interact with certain class I HLA molecules (which can function as KIR ligands) to modulate NK cell responses [16, 17]. The interactions between endogenous KIRs and KIR-ligands modulate NK cell function and immunotherapeutic responses [12, 18–21]. During NK cell maturation, NK cells lacking inhibitory KIRs specific for self-HLA class I become less potent than NK cells expressing one or more inhibitory receptors for self-HLA class I through a process termed licensing [22, 23]. Yet, these same inhibitory KIRs can suppress mature NK cells through specific interactions with the class I HLA molecules that function as their ligands when expressed by tumors and other nucleated cells.

Prior clinical studies have reported associations between KIR/KIR-ligand genotypes and patient clinical response in various immunotherapeutic settings that likely involve NK cells [12, 18, 20, 24–29]. In a separate randomized clinical trial of patients with neuroblastoma, we have shown that patients with certain KIR/KIR-ligand genotypes benefited from treatment with mAb-based immunotherapy [dinutuximab (anti-GD2 mAb) + GM-CSF + interleukin-2] while patients with the opposing KIR/KIR-ligand genotype were not clinically influenced by the immunotherapy treatment [30]. In addition, we found that patients that did *not* receive immunotherapy responded differently dependent upon which KIR/KIR-ligand genotypes they had, suggesting that NK cells influence outcome even in the absence of immunotherapy [30].

In this study, we assessed whether the presence of certain KIR/KIR-ligand genotypes might affect whether the use of maintenance rituximab influences outcome. Separately, we also investigated whether certain KIR/KIR-ligand genotypes influenced the clinical outcome based on the rituximab treatment schedule (i.e. how KIR/KIR-ligand genotype might affect clinical outcome within the maintainance or non-maintenance treatment schedules). The primary endpoint in the E4402 clinical trial was the time to rituximab failure (TTRF). In this present study, we evaluated associations of KIR/KIR-ligand genotype with TTRF, using determinants based on biological causes (detailed in the statistical section of Methods). We also assessed two additional biologically relevant clinical parameters: duration of response and % tumor shrinkage. In the current report, we found that for subsets of patients with certain KIR/KIR-ligand genotypes (but not for others), clinical outcome (TTRF) was improved by the maintenance rituximab treatment schedule.

## Methods

### Clinical trial and clinical samples

The phase III ECOG-ACRIN E4402 clinical trial (ClinicalTrials.gov #NCT00075946) evaluated the efficacy of single agent, rituximab therapy for adults with low-tumor burden indolent lymphoma. Clinical results from this study have been reported elsewhere [6]. A total of 408 patients with follicular lymphoma were entered, 289 of which responded and were randomized to maintenance vs non-maintenance therapy with rituximab. Disease measurements were obtained every 13 weeks [6]. Of the 408 patients, 213 patients had evaluable DNA and clinical data; of the 289 responding randomized patients, 159 had evaluable DNA and clinical data for this study. Clinical and demographic data for the 213 patients and the 159 randomized patients for which DNA and clinical data were evaluable (non-maintenance *n* = 80 and maintenance *n* = 79) are included in Additional file 1: Table S1, along with comparative data for the 289 responding patients reported on in the primary clinical report. The clinical trial was conducted in accordance with the Helsinki Declaration of 1975.

### Genotyping

KIR gene status was determined by a real time PCR technique [31, 32]. The genotypes of KIR-ligand (HLA-C1, HLA-C2, HLA-Bw4) were performed in a blinded fashion, and determined by PCR-SSP using the KIR HLA Ligand SSP typing kit (Olerup) with GoTaq DNA Polymerase (Promega). Additional genotyping details can be found in the supplemental methods section.

### Data management

Study data (genotyping data from our lab) were entered into and managed using the REDCap system hosted at the University of Wisconsin-Madison. REDCap (Research Electronic Data Capture) is a secure, HIPAA-compatible, web-based application designed to support data capture for research studies, providing: 1) an intuitive interface for validated data entry; 2) audit trails for tracking data manipulation and export procedures; and 3) procedures for importing data from external sources [33]. The clinical outcome data from the ECOG-ACRIN study database (which is HIPAA compliant) in Excel were merged with the genotyping data in REDCap to create a SAS dataset for analysis***.***

### KIR/KIR-ligand interaction analysis

Individuals that have all KIR-ligands present for the inhibitory KIRs they possess were defined as having a “KIR-ligands present” genotype. Individuals that lack any KIR-ligand for any one of the KIR genes they possess were defined as having a “KIR-ligand missing” genotype [18, 20, 29, 30]. Detailed descriptions of these genotypes can be found in Additional file 1: Table S2.

### Statistical methods

The primary objective was to evaluate the association of clinical outcome with treatment regimen and KIR-ligand status (all KIR-ligands present compared with KIR-ligands missing). Other analyses were exploratory, but the KIR/KIR-L genotype combinations evaluated here were performed based on associations with outcome for similar KIR/KIR-L genotype combinations in a prior study of neuroblastoma patients treated with a separate mAb [30]. Thus, statistical analyses were performed, and *p* values are reported, without any adjustment for multiplicity of testing. Only randomized patients were included in the analyses. The post-hoc analysis of the clinical outcomes from this phase III trial that were assessed included the duration of response (*n* = 155), the time to rituximab failure (TTRF) (*n* = 159), and % tumor shrinkage (*n* = 139). For each of the clinical parameters assessed, Additional file 1: Table S3 includes the mean/median response data with 95% confidence intervals. The duration of response was defined as the time from randomization (following an initial response to the induction rituximab treatment) to documented first disease progression. The TTRF was defined as the time from randomization to treatment failure, as reported by Kahl et al. [6]. Treatment failure was defined as whichever came first of: 1) the time at which patients no longer responded to rituximab [disease progression for patients receiving maintenance; no response to retreatment rituximab or time to progression [< 26 weeks from day 1 of last rituximab for patients in the non-maintenance treatment schedule], 2) the time at which an alternative therapy was initiated, or 3) the time at which patients were determined unable to complete their assigned rituximab schedule [6]. For all TTRF analyses reported here, treatment failures that were considered non-biological (largely the decision to change to alternate therapy by the patient or physician) were censored, and as presented in the supplemental materials in the initial clinical report [6].

The % tumor shrinkage was defined as the % change in tumor size from the baseline measurement at the time of randomization (measured 13 weeks after initiating the induction rituximab treatment) to the smallest tumor size obtained post-randomization, only including those who had a partial response 13 weeks after initiating the induction rituximab (*n* = 139). While all evaluable randomized patients could be analyzed for duration of response, tumor shrinkage following randomization could not be calculated for 16 patients (10 in non-maintenance and 6 in maintenance) that had achieved a complete response with a tumor measurement of zero at week 13 of induction, as it was impossible to measure any further shrinkage after their complete response measured at week 13. Changes in tumor size were represented using box plots (described in detail in supplemental materials).

Log-rank tests and Cox proportional hazards regression models were used to compare the duration of response and TTRF by treatment and genotype combinations. For the % tumor shrinkage, analysis of variance (ANOVA) was used. For our analyses, the associations between outcome and KIR/KIR-ligand were evaluated using Cox regression models with treatment group and KIR/KIR-ligand genotype as the main effects. In addition, we evaluated for possible interaction effects between treatment schedule and KIR/KIR-ligand genotype on outcome. For the analyses assessing associations of outcome with specific KIRs and their ligands (as in Tables 1 and 2), we set a minimal *p*-value of *p* < 0.100 requirement in the interaction analysis in order to subsequently perform association comparisons directly between individual genotype groups and outcome. Statistical analyses were performed using SAS v9.4 (SAS Institute, Cary, NC).

**Table 1:** Interaction analyses for individual KIR and KIR ligand genotypes with TTRF

			TTRF		
Line	Treatment	Genotype Group	Number of Events/n	4 yr Fail Rate (95% CI)^a^ %	Interaction *p*-value
1	Maintenance	KIR2DL1+/C2+	21/52	38 (25–54)	0.524
2	Maintenance	*not* KIR2DL1+/C2+	7/28	30 (15–56)	
3	Non-Maintenance	KIR2DL1+/C2+	18/49	38 (26–55)	
4	Non-Maintenance	*not* KIR2DL1+/C2+	6/30	21 (9–43)	
5	Maintenance	KIR2DL2+/C1+	11/36	32 (18–53)	0.547
6	Maintenance	*not* KIR2DL2+/C1+	17/44	38 (25–56)	
7	Non-Maintenance	KIR2DL2+/C1+	6/28	24 (10–49)	
8	Non-Maintenance	*not* KIR2DL2+/C1+	18/51	36 (24–52)	
9	Maintenance	KIR2DL3+/C2+	17/56	33 (21–50)	0.982
10	Maintenance	*not* KIR2DL3+/C2+	11/24	41 (23–64)	
11	Non-Maintenance	KIR2DL3+/C2+	16/60	27 (17–43)	
12	Non-Maintenance	*not* KIR2DL3+/C2+	8/19	45 (25–70)	
13	**Maintenance**	**KIR3DL1+/Bw4+**	**20/54**	**38 (25–55)**	**0.055**
14	**Maintenance**	***not*** **KIR3DL1+/Bw4+**	**8/26**	**29 (15–52)**	
15	**Non-Maintenance**	**KIR3DL1+/Bw4+**	**13/50**	**25 (15–41)**	
16	**Non-Maintenance**	***not*** **KIR3DL1+/Bw4+**	**11/29**	**43 (25–66)**	

^a^95% Confidence interval; lines 13-14 (bolded text) had a *p*-value <0.100 and were analyzed further for associations with outcome

**Table 2:** Interaction analyses for double-inhibitory KIR and KIR ligand genotypes with TTRF

			TTRF		
Line	Treatment	Genotype Group	Number of Events/n	4 yr Fail Rate (95% CI)^a^ %	Interaction *p*-value
1	Maintenance	KIR2DL1+/C2+ and KIR3DL1+/Bw4+	17/44	37 (24–54)	0.694
2	Maintenance	*not* KIR2DL1+/C2+ and KIR3DL1+/Bw4+	11/36	34 (19–55)	
3	Non-Maintenance	KIR2DL1+/C2+ and KIR3DL1+/Bw4+	10/32	33 (18–54)	
4	Non-Maintenance	*not* KIR2DL1+/C2+ and KIR3DL1+/Bw4+	14/47	31 (19–48)	
5	**Maintenance**	**Group 1: KIR2DL2+/C1+ and KIR3DL1+/Bw4+**	**9/22**	**45 (24–72)**	**0.068**
6	**Maintenance**	**Group 2:** ***not*** **KIR2DL2+/C1+ and KIR3DL1+/Bw4+**	**19/58**	**32 (21–47)**	
7	**Non-Maintenance**	**Group 1: KIR2DL2+/C1+ and KIR3DL1+/Bw4+**	**4/23**	**17 (6–44)**	
8	**Non-Maintenance**	**Group 2:** ***not*** **KIR2DL2+/C1+ and KIR3DL1+/Bw4+**	**20/56**	**38 (26–54)**	
9	Maintenance	KIR2DL3+/C1+ and KIR3DL1+/Bw4+	12/35	38 (26–54)	0.447
10	Maintenance	*not* KIR2DL3+/C1+ and KIR3DL1+/Bw4+	16/45	34 (21–51)	
11	Non-Maintenance	KIR2DL3+/C1+ and KIR3DL1+/Bw4+	10/37	25 (13–44)	
12	Non-Maintenance	*notT* KIR2DL3+/C1+ and KIR3DL1+/Bw4+	14/42	38 (24–57)	

^a^95% Confidence interval; lines 5-8 (bolded text) had a *p*-value <0.100 and were analyzed further for associations with outcome

## Results

### KIR ligand missing status does not significantly influence TTRF, duration of response or tumor shrinkage

**Fig. 1:**
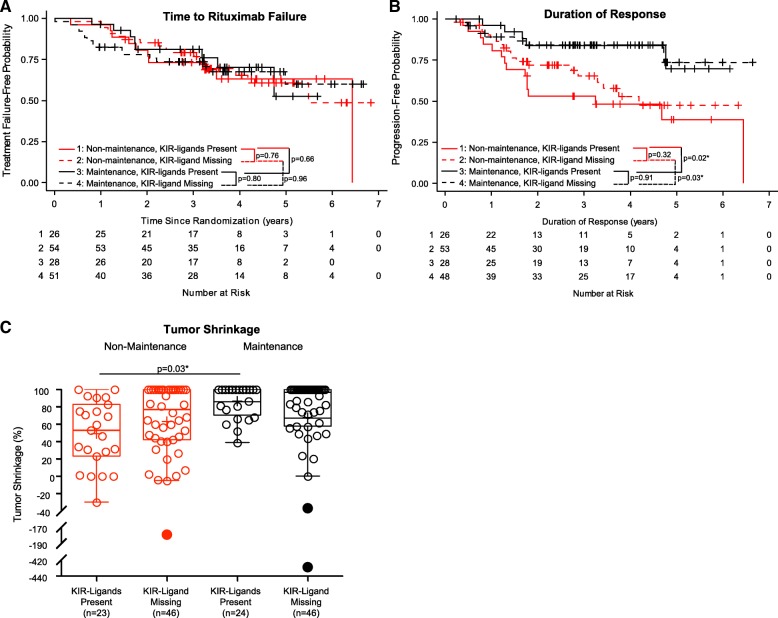
Associations of overall KIR/KIR-ligand status with clinical outcomes. Kaplan-Meier curves for TTRF (non-biological events censored) (**a**) and for duration of response (**b**) compare those treated with maintenance rituximab and KIR-ligands present (Line 1: solid-black line), those treated with maintenance rituximab and KIR-ligand missing (Line 2: dashed-black line), those treated with non-maintenance rituximab and KIR-ligands present (Line 3: solid-red line) and those treated with non-maintenance rituximab and KIR-ligand missing (Line 4: dashed-red line). **c** displays box-plots for % tumor shrinkage for the four groups above (*p*-value not shown if *p* > 0.1; “*” indicates *p* < 0.05). Outlying values are shown as filled circles outside the horizontal lines

In some previous studies, patients with at least one KIR-ligand missing (“KIR-ligand missing”) had improved clinical outcome as compared to those with all KIR- igands present (“KIR-ligands present”) when treated with NK-based immunotherapy [18, 25, 26, 29]. The genotypes used to define KIR-ligands present vs. KIR-ligand missing are detailed in Additional file 1: Table S2. Based on the findings from these prior studies, we hypothesized that FL patients that received rituximab therapy that had the KIR-ligand missing genotype would have improved outcome as compared to those patients with KIR-ligands present. However, here we found no significant association between TTRF with KIR-ligands present vs. KIR-ligand missing status (Fig. 1a) amongst either those receiving maintenance or those receiving non-maintenance. Similarly, KIR-ligands present vs. KIR-ligand missing status did not influence duration of response (Fig. 1b) or tumor shrinkage (Fig. 1c) for either treatment regimen. In addition, the treatment regimen (maintenance vs. non-maintenance) did not influence TTRF for patients with either KIR-ligands present or KIR-ligand missing (Fig. 1a), consistent with the data for the overall group of patients from the initial clinical report [6]. In contrast, amongst patients with KIR ligands present, those receiving maintenance showed significantly increased duration of response (Fig. 1b) and tumor shrinkage (Fig. 1c), and those with KIR ligands missing that received maintenance also showed increased duration of response (Fig. 1b).

### Specific individual inhibitory KIR/KIR-ligand combinations: The presence of KIR3DL1 and its HLA-Bw4 ligand, but not absence of KIR3DL1 and HLA-Bw4, are associated with improved outcome for patients receiving rituximab maintenance treatment

We hypothesized that patients with all KIR-ligands present have NK cells that are more inhibited due to the presence of KIR-ligands for all of the inhibitory KIRs inherited [18, 20]. Yet, there is also the concept that those patients with KIR-ligands present have more licensed NK cells that may be able to better lyse rituximab-treated tumor cells [22, 34, 35]. We further assessed whether there was a differential influence on outcome in this clinical trial dependent upon the presence or absence of specific inhibitory KIRs/KIR-ligands.

HLA-C alleles can be divided based on their KIR binding as HLA-C1 or HLA-C2 containing epitopes [19, 36]. As C1 and C2 are alleles, every individual will genotype as either HLA-C1/C1, HLA-C1/C2, or HLA-C2/C2. KIR2DL1 recognizes HLA-C2 as its ligand. Thus individuals with both KIR2DL1 and HLA-C2 (designated as KIR2DL1+/C2+) include those that have KIR2DL1+ and also have HLA-C2+ (HLA-C genotype of either C1/C2 or C2/C2). All other possible genotypes regarding KIR 2DL1 and HLA-C are designated as *not* KIR2DL1+/C2+ and include the following genotypes: (KIR2DL1+/C2-, KIR2DL1−/C2+, or KIR2DL1−/C2-) described in detail in Additional file 1: Table S4. KIRs 2DL2 and 2DL3 each recognize HLA-C1 as their KIR-ligand. Thus KIR2DL2+/C1+ individuals have KIR2DL2+ with HLA-C1+ (HLA-C genotype of either C1/C1 or C1/C2); all other possible genotypes of KIR2DL2 and HLA-C are designated as *not* KIR2DL2+/C1+, as detailed in Additional file 1: Table S4. Similarly, KIR2DL3+/C1+ individuals have KIR2DL3+ with HLA-C1+ (HLA-C genotype of C1/C1 or C1/C2); all other KIR2DL3 and HLA-C genotypes are designated as *not* KIR2DL3+/C1+ (Additional file 1: Table S4). KIR3DL1 recognzies the HLA-Bw4 epitope on HLA-B and HLA-A. Thus KIR3DL1+/Bw4+ individuals include all those that have KIR3DL1+ with either HLA-B-Bw4+ and/or HLA-A-Bw4+ [37]. All other KIR3DL1 and HLA-Bw4 genotypes are designated as *not* KIR3DL1+/Bw4+ (Additional file 1: Table S4).

**Fig. 2:**
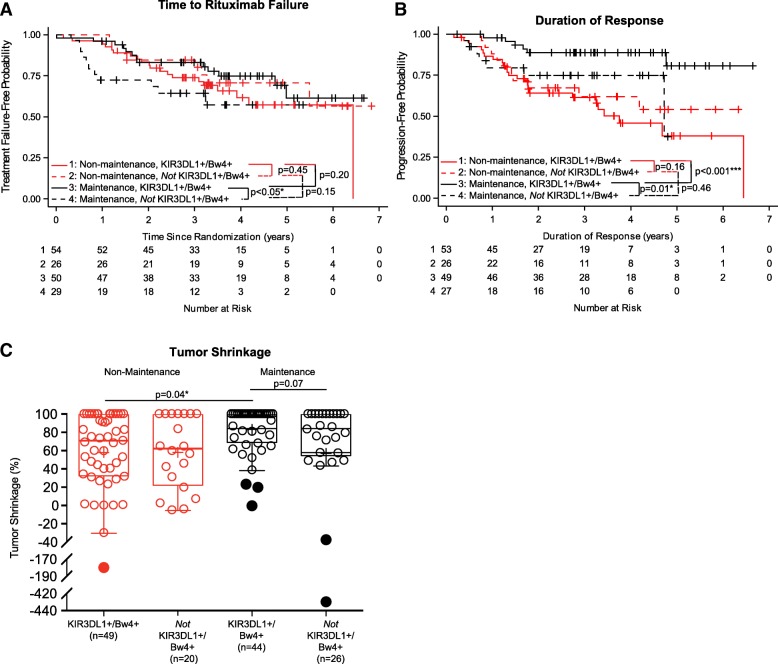
Associations of KIR3DL1 and its ligand status with clinical outcomes. Kaplan-Meier curves for TTRF (non-biological events censored) (**a**) and for duration of response (**b**) compare those treated with maintenance rituximab and KIR3DL1+/Bw4+ (Line 1: solid-black line), those treated with maintenance rituximab and *not* KIR3DL1+/Bw4+ (Line 2: dashed-black line), those treated with non-maintenance rituximab and KIR3DL1+/Bw4+ (Line 3: solid-red line) and those treated with non-maintenance rituximab and *not* KIR3DL1+/Bw4+ (Line 4: dashed-red line). **c** displays box-plots for percent tumor shrinkage for the four groups above (*p*-value not shown if *p* > 0.1). Outlying values are shown as filled circles outside the horizontal lines. (“*” indicates *p* < 0.05; “***” indicates *p* < 0.001)

To assess whether the effect of individual KIR/KIR-ligand pairs may be influenced by treatment regimen, we first did an interaction analysis between outcome for each treatment regimen and genotype status for individual KIR/KIR-ligand pairs (Table 1). Since TTRF was the primary endpoint in the E4402 clinical trial [6], for the initial interaction analysis, we used TTRF as our primary endpoint. Only the genotypes determined by KIR3DL1/Bw4 showed an interaction *p*-value ≤0.100 with treatment regimen (corresponding to lines 13–16 in Table 1). This *p*-value of 0.055 justified further analyses of associations of outcome (shown in Fig. 2) with these 4 individual KIR/KIR-ligand groups.

For patients that were KIR3DL1+/Bw4+, those that received the maintenance regimen showed significantly improved duration of response (*p* < 0.001, Fig. 2b) and % tumor shrinkage (*p* = 0.04, Fig. 2c) vs. those that did not receive maintenance. There was no significant improvement in duration of response or tumor shrinkage associated with maintenance treatment vs. non-maintenance for the patients that were *not* KIR3DL1+/Bw4+. These results suggest that the maintenance regimen improved clinical outcome for KIR3DL1+/Bw4+ patients. Additionally, amongst patients receiving maintenance rituximab, KIR3DL1+/Bw4+ patients had improved outcome compared to those that were *not* KIR3DL1+/Bw4+ (*p* < 0.05 for TTRF, Fig. 2a; *p* = 0.01 for duration of response, Fig. 2b; and a trend of *p* = 0.07 for tumor shrinkage, Fig. 2c).

### Inhibitory KIR2DL2/C1+ interactions in combination with KIR3DL1/KIR-ligand interactions improve outcome for patients receiving rituximab maintenance treatment

While the KIR-ligands present genotype (as shown in Fig. 1) considers all three KIR-ligands (HLA-C1, HLA-C2 and HLA-Bw4) equally contributing to the licensing or inhibition of NK cells, (described in Additional file 1: Table S2), we assessed whether certain combinations of KIR2DL1, KIR2DL2 or KIR2DL3 and their KIR-ligands together with KIR3DL1 and its KIR-ligand would affect patients’ response to rituximab differently. We and others have found that a subset of patients, based on the presence of KIR2DL1, KIR2DL2 or KIR2DL3 and their respective ligands together with the presence of KIR3DL1 and its ligand, benefited from anti-GD2-based treatment while the converse genotype did not [38, 39]. As mentioned above, HLA-C1 and HLA-C2 are alleles, and thus everyone will have either HLA-C1 and/or HLA-C2. With this in mind, since every individual will have an interaction of inhibitory KIR2DL1, KIR2DL2 or KIR2DL3 with their HLA-C ligands, we assessed the effects of the presence or absence of KIR3DL1 and its ligand on outcome in combination with the various possible combinations of KIR2DL1, KIR2DL2 or KIR2DL3 with their ligands.

The following dual combinations of inhibitory KIRs were compared:

KIR2DL1+/C2+/KIR3DL1+/Bw4+ vs. *not* KIR2DL1+/C2+/KIR3DL1+/Bw4+; KIR2DL2+/C1+/KIR3DL1+/Bw4+ vs. *not* KIR2DL2+/C1+/KIR3DL1+/Bw4+;and KIR2DL3+/C1+/KIR3DL1+/Bw4+ vs. *not* KIR2DL3+/C1+/KIR3DL1+/Bw4. These double-inhibitory KIR/KIR-ligand genotype combinations are described in further detail in Additional file 1: Table S5. For these “double” combinations, the only interaction comparison that met our predefined statistical cutoff of a *p*-value ≤0.100 was the comparison of KIR2DL2+/C1+/KIR3DL1+/Bw4+ vs. *not* KIR2DL2+/C1+/KIR3DL1+/Bw4+, herein referred to as “Group 1” vs. “Group 2”, respectively (as shown in Table 2, lines 5–9, *p* = 0.068).

**Fig. 3:**
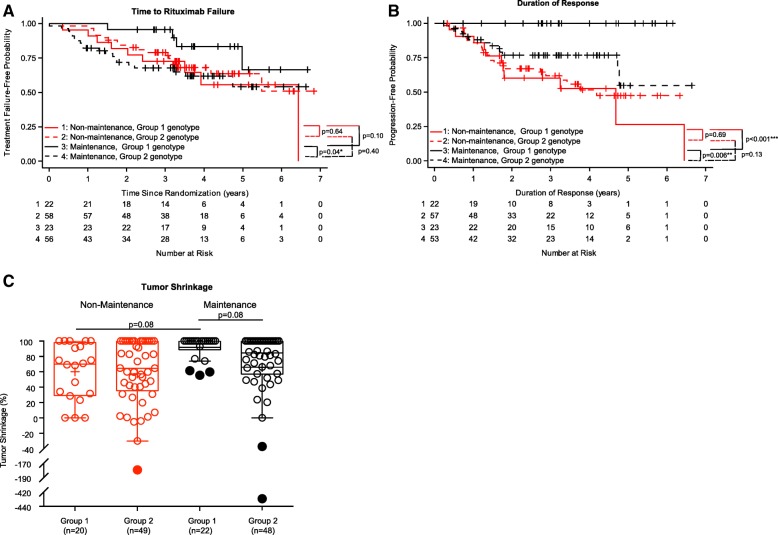
Associations of KIR2DL2 and KIR3DL1 and their ligand status with clinical outcomes. Kaplan-Meier curves for TTRF (non-biological events censored) (**a**) and for duration of response (**b**) compare those treated with maintenance rituximab and Group 1 (KIR2DL2+/C1+/KIR3DL1+/Bw4+) (Line 1: solid-black line), those treated with maintenance rituximab and Group 2 (*not* KIR2DL2+/C1+/KIR3DL1+/Bw4+) (Line 2: dashed-black line), those treated with non-maintenance rituximab and Group 1 (Line 3: solid-red line) and those treated with non-maintenance rituximab and Group 2 (Line 4: dashed-red line). **c** displays box-plots for percent tumor shrinkage for the four groups above (*p*-value not shown if *p* > 0.1). Outlying values are shown as filled circles outside the horizontal lines. (“*” indicates *p* < 0.05; “***” indicates *p* < 0.001)

Subgroup comparisons were done for Group 1 vs. Group 2 treated with maintenance vs. non-maintenance rituximab (Fig. 3). Group 1 patients treated with maintenance vs. non-maintenance showed a trend toward improved TTRF (*p* = 0.10, Fig. 3a), improved duration of response (*p* < 0.001, Fig. 3b), and a trend toward improved % tumor shrinkage (*p* = 0.08, Fig. 3c). Conversely, those in Group 2 did not even have a trend toward improved clinical outcome for any of these 3 parameters if treated with maintenance vs. non-maintenance rituximab (Fig. 3a, b and c). In addition, amongst patients that were treated with maintenance rituximab, Group 1 had improved clinical outcome as compared to those in Group 2 (*p* = 0.04 for TTRF, Fig. 3a; *p* = 0.006 for duration of response, Fig. 3b; *p* = 0.08 for tumor shrinkage, Fig. 3c).

## Discussion

In this study, we found no influence of the KIR-ligands present vs. KIR-ligand missing genotypes on clinical outcome. However, we did find that for some FL patients, treatment with maintenance rituximab improved their clinical outcome based on their KIR/KIR-ligand genotype. Namely, the 28% (45 of 159) of this patient population with genotypes in Group 1 (Fig. 3a) appear to have improved outcome (significant for duration of response and tumor shrinkage and a trend for TTRF) from maintenance vs. non-maintenance rituximab. In contrast, for those remaining 72% of patients with the Group 2 genotype, we found insufficient evidence of an improvement in outcome if treated with maintenance compared to the non-maintenance rituximab treatment schedule [18, 20]. We recently found a similar result for these same genotypes in a separate randomized study of anti-GD2-based mAb immunotherapy for children with high-risk neuroblastoma. Namely, neuroblastoma patients with the Group 1 genotype appear to have improved outcome from anti-GD2-based immunotherapy (vs. no immunotherapy), while for those patients with the Group 2 genotype, no improvement in outcome from the anti-GD2-based immunotherapy (vs. no immunotherapy) was observed [38]. These similar results regarding improved outcome with the immunotherapy regimens for patients with the Group 1 genotype in these two separate randomized studies, [which include two different patient poluations (adult vs. pediatric patients), two different disease types (FL vs. neuroblastoma), and two different anti-tumor-reactive mAb-based immunotherapies (rituximab/anti-CD20 vs. dinutuximab/anti-GD2)], provides some degree of validation for this finding. In addition to the Group 1 genotype, we recently reported similar findings for patients with certain HLA-Bw4 isoforms from these same two clinical studies [40]. In that report, we evaluated the role of three separate HLA-Bw4 isoforms (HLA-A-Bw4, HLA-B-Bw4-I80 and HLA-B-Bw4-T80). In both clinical trials, patients with KIR3DL1+/A-Bw4+ or with KIR3DL1+/B-Bw4-T80+ genotypes randomized to neuroblastoma immunotherapy or FL maintenance immunotherapy had better outcome versus those randomized to no immunotherapy for neuroblastoma or non-maintenance for FL. In contrast, for those with KIR3DL1+/B-Bw4-I80+, there was no evidence of a difference in outcome between immunotherapy vs. no-immunotherapy [40]. These combined results suggest that the correlation between these KIR/KIR-ligand genotypes and outcome may serve as a biomarker for identifying those that might benefit from this type of immunotherapy using tumor-reactive mAb. Furthermore, these results suggest similar analyses of KIR/KIR-ligand genotype and immunotherapy should be pursued for other clinical trials in other diseases that utilize other tumor-reactive mAbs to see if these findings we have noted for rituximab in FL and dinutuximab in neuroblastoma might extend to other cancers treated with other tumor-reactive mAb [40].

In addition, in this study, amongst patients that received rituximab maintenance, those that are KIR3DL1+/Bw4+ showed significantly improved TTRF, duration of response and tumor shrinkage than for those *not* KIR3DL1+/Bw4+. Similarly, amongst patients that received rituximab maintenance, those that are Group-1 (KIR2DL2+/C2+ and KIR3DL1+/Bw4+) showed improved TTRF, duration of response and tumor shrinkage than those that are Group-2 (*not* KIR2DL2+/C2+ and KIR3DL1+/Bw4+). These demonstrate an association (likely via ADCC) of KIR/KIR-ligand genotype on the outcome of rituximab maintenance therapy.

Several studies have found associations of patient response to treatment with KIR2DL2 status, with or without its ligand, HLA-C1 [41–43]. In a study of neuroblastoma patients mentioned previously, we found that patients that had both KIR2DL2+/C1+ [KIR2DL2+ and HLA-C1+ (C1/C1 or C1/C2)] and KIR3DL1+/Bw4+ (i.e. Group 1) had improved clinical outcome if treated with monoclonal antibody-based immunotherapy compared to no-immunotherapy [38].

In addition to assessing the influence of HLA-Bw4 on clinical outcome, Du and colleagues found that FL patients that were missing both HLA-C2 and HLA-Bw4 and were treated with either rituximab and galaximib, or rituximab and epratuzamab, had longer duration of response than those individuals that possessed both of those KIR-ligands [12]. That analysis focused on the subsets of patients with both HLA-C2 and HLA-Bw4 or missing both HLA-C2 or HLA-Bw4, but excluded the subsets with only one or the other of these combinations. In our analysis here of FL patients treated with maintenance or non-maintenance rituximab, we included all patients in each comparative analysis, and also considered the inhibitory KIR gene status, when evaluating KIR2DL1, KIR2DL2 or KIR2DL3, with their respective ligands with KIR3DL1 and its HLA-Bw4 ligand. With respect to HLA-C status and HLA-Bw4 status, following assessment of all possible KIR2DL1, KIR2DL2 or KIR2DL3, with their respective ligands (HLA-C1 or HLA-C2) and with KIR3DL1 and its ligand (HLA-Bw4), our analysis suggests patients that are both KIR2DL2+/C1+ and KIR3DL1+/Bw4+ (Group 1) had improved clinical outcome if treated with rituximab maintenance compared with the non-maintenance treatment schedule. However, as the subpopulations of patients with varying KIRs and KIR-ligands studied in each report are different, and the exact therapy we used (rituximab maintenance) was different than that used by Du et al. (rituximab and galaximib, or rituximab and epratuzamab), we cannot actually determine whether our results are discordant from those of Du et al.

Kahl and colleagues used TTRF as their primary endpoint in the primary clinical report of this trial, as it may provide more value to understanding of the clinical benefit of rituximab than using duration of response as an endpoint [6, 8]. Rituximab has limited side effects compared with cytotoxic chemotherapeutic agents, which have more adverse side effects [8], and the TTRF can reflect when progressive or unresponsive disease requires initiation of cytotoxic chemotherapy. If a maintenance schedule can, for some patients, delay the need for cytotoxic therapy, this could provide clinical benefit [44, 45]. In our analyses of the TTRF parameter shown in this report, we considered only those failure events that were considered to be biologically relevant. In the primary study report, Kahl et al. noted no difference in time to rituximab failure between the maintenance and non-maintenance schedules when all patients were evaluated independent of genotype [6]. However, in this report, by assessing different genotype groupings of KIRs/KIR-ligands, we were able to identify a set of patients (Group 1: KIR2DL2+/C1+/KIR3DL1+/Bw4+), reflecting 28% of this patient population, that may have prolonged TTRF if treated with maintenance rituximab as compared to non-maintenance. This finding provides some evidence that rituximab maintenance may still provide a clinically meaningful benefit for a subgroup of patients based on their KIR/KIR-ligand genotypes.

Most NK cells express an array of both inhibitory and activating receptors; NK cell activation reflects the balance of both activating and inhibitory signals. The clinical data and associations presented in this report suggest that there are in vivo interactions that were simultaneously influenced by inhibitory KIRs and their ligands, and activating signaling through stimulation with mAb (rituximab), which appeared to also reflect influences on NK cell licensing [35]. While this report focuses on the associations of inhibitory-KIR/KIR-ligand genotype with clinical outcome for this clinical trial, patients from this same clinical trial were also evaluated for other KIR-related genotype associations. We also evaluated associations of KIR haplotype (A or B) with clinical outcome, but found no associations with clinical outcome (data not shown) [24]. In addition, for this same ECOG-ACRIN study, Kenkre et al. assessed genotypes for high and low affinity allelic variants of the activating Fc gamma receptors (FCGR), FCGR3A and FCGR2A, and found no associations with clinical outcome [46].

## Conclusions

In summary, the associations of outcome and KIR/KIR-ligand genotypes presented here demonstrate some role for KIR recognition of KIR-ligands in the in vivo response to rituximab therapy in at least some patients with FL. In particular, the 28% of patients in this trial that have the Group 1 genotype had an association of clinical outcome benefit with administration of the rituximab maintenance treatment schedule. Whether the statistically significant benefit in outcome for this subset shown here merits prospective testing (via genotyping of all patients to identify this group for further analyses of maintenance treatment) requires consideration of medical as well as cost-related issues. Furthermore, as the associations observed here are based on relatively small numbers of patients in the individual comparative groups, separate validation may be helpful before considering whether this KIR/KIR-ligand genotyping should be used prospectively for treatment assignment decisions. In addition, based on the findings described here, as well as our similar findings using other monoclonal antibodies [30], it may be of interest to study the impact of KIR and KIR-ligands with the newer anti-CD20 antibodies, obinutuzumab and ofatumumab, which have shown efficacy in treatment of indolent non-Hodgkin lymphomas [47, 48]. Finally, the associations reported here likely reflect the regulation of NK cells mediating in vivo ADCC as a result of the rituximab therapy, consistent with other preclinical and clinical data. Thus these data further support the roles that KIR and KIR-ligands play, for at least some patients, in the setting of certain types of cancer immunotherapy.

## Additional file

Additional file 1: **Table S1.** Description of KIR/KIR-ligand genotypes included in analyses for Fig. 1. **Table S2.** Description of KIR/KIR-ligand genotypes included in analyses for Table 1 and Fig. 2. **Table S3.** Detailed statistical information for data pertaining to Figs. 1, 2 and 3. **Table S4.** Description of KIR/KIR-ligand genotypes included in analyses for Table 2 and Fig. 3. **Table S5.** Demographic and clinical characteristics of ECOG patients with. FL that were randomly assigned and genotyped. (DOCX 531 kb)

## Abbreviations

ECOG-ACRIN: ECOG-ACRIN Cancer Research Group
FCGR: Fc gamma receptors
FL: Follicular-lymphoma
Group 1: KIR2DL2+/C1+/KIR3DL1+/Bw4+
Group 2: *not* KIR2DL2+/C1+/KIR3DL1+/Bw4+
KIR: Killer immunoglobulin-like receptors
Maintenance: Rituximab every 13 weeks
NK Cells: Natural Killer cells
Non-Maintenance: No additional rituximab until progression
TTRF: Time to Rituximab Failure
