# Supplementary material for: Follicular lymphoma patients with KIR2DL2 and KIR3DL1 and their ligands (HLA-C1 and HLA-Bw4) show improved outcome when receiving rituximab
Source: J Immunother Cancer. 2019 Mar 12;7:70. doi: 10.1186/s40425-019-0538-8 (PMC6419437; doi:10.1186/s40425-019-0538-8)
Supplement: Supplementary file 1 — Table S1. Description of KIR/KIR-ligand genotypes included in analyses for Fig. 1. Table S2. Description of KIR/KIR-ligand genotypes included in analyses for Table 1 and Fig. 2. Table S3. Detailed statistical information for data pertaining to Figs. 1, 2 and 3. Table S4. Description of KIR/KIR-ligand genotypes included in analyses for Table 2 and Fig. 3. Table S5. Demographic and clinical characteristics of ECOG patients with. FL that were randomly assigned and genotyped. (DOCX 531 kb) [file 40425_2019_538_MOESM1_ESM.docx]

**SUPPLEMENTAL MATERIALS**

**Supplemental Materials and Methodology**

**Clinical Trial and Clinical Samples:**

As duration of response is defined as the time from randomization to first progression, and non-maintenance patients received no additional rituximab treatment after randomization until their first progression, the duration of response parameter for non-maintenance patients describes the longevity of their response following the initial 13 weeks of induction therapy, without any subsequent rituximab treatment prior to progression. All relevant medicine agencies and ethics committees approved the study protocol. Informed consent was obtained from all patients [6].

**Genotyping Validation:**

Our genotyping methodology was validated in a separate clinical neuroblatoma study [30], where we used the same genotyping method as used in this ECOG study. For that neuroblastoma study, we received 251 DNA samples from two separate sites. Among those 251 samples, there were 60 patients from which we received duplicate DNA samples, labeled separately, with one from each site. Those samples were received and genotyped blindly. Genotyping results were then compared between those duplicate samples for these 60 patients. Of a total of 900 KIR genes we genotyped for the 60 patients with duplicate samples, there were 10 KIR genes for which we have discrepant results (KIR discrepancy rate: 1.1%). Among the total of 360 HLA genes that we genotyped for the 60 patients, we had discrepant results for 5 HLA genes (HLA discrepancy rate: 1.4%) [30].

**Box Plots:**

Changes in tumor size were represented using box plots, which show the 25th percentile (Q1) (bottom of box), the 50th percentile (Q2) (bolded black line), the 75th percentile (Q3) (top of box), and the mean (red cross inside the box). The lower and upper short horizontal red lines represent the minimum and maximum values, excluding the outlying high and low values. Outlying values [i.e.: those that are a distance of more than 1.5x(Q3-Q1) from the box], are shown as circles outside the horizontal lines.

**Supplemental Tables**

**Supplemental Table 1. Description of KIR/KIR-ligand genotypes included in analyses for Figure 1.**

**Supplemental Table 2. Description of KIR/KIR-ligand genotypes included in analyses for Table 1 and Figure 2.**

**Supplemental Table 3:**  **Detailed statistical information for data pertaining to Figures 1, 2 and 3.** In Column A, for each clinical outcome measure, the values for the separate treatment and genotype groups are compared to the index value “#” [to consider the “Genotype Effect” (e.g. for those treated with Maintenance, compare KIR-ligands Present vs. KIR-ligand Missing) or the “Treatment Effect” (e.g. for those with KIR-ligands Present, compare Maintenance vs. Non-Maintenance)].  In Column B, for each clinical outcome measure, the values for the separate treatment and genotype groups are compared to the index value “$” [to consider the “Genotype Effect” (e.g. for those treated with Non-Maintenance, compare KIR-ligands Present vs. KIR-ligand Missing) or the “Treatment Effect” (e.g. for those with KIR-ligand Missing, compare Maintenance vs. Non-Maintenance)]. In Columns A and B, “NA” represents comparisons that are “Not Applicable” as there is no common variable within those cells of the table to assess a Treatment or Genotype effect (i.e. “Maintenance with KIR-Ligands Present” cannot be directly compared to “Non-Maintenance with KIR-Ligand Missing”).

**Supplemental Table 4. Description of KIR/KIR-ligand genotypes included in analyses for Table 2 and Figure 3.**

**Supplemental Table 5. Demographic and clinical characteristics of ECOG patients with**

**FL that were randomly assigned and genotyped.**
